# Periostin Is a Biomarker of Rheumatoid Arthritis-Associated Interstitial Lung Disease

**DOI:** 10.3390/jcm12227100

**Published:** 2023-11-15

**Authors:** Goushi Matama, Masaki Okamoto, Kiminori Fujimoto, Takeshi Johkoh, Masaki Tominaga, Hiroshi Mukae, Noriho Sakamoto, Kosaku Komiya, Kenji Umeki, Masamichi Komatsu, Yasuo Shimizu, Koichiro Takahashi, Saeko Tokisawa, Yoshiaki Zaizen, Norikazu Matsuo, Takashi Nouno, Shinjiro Kaieda, Hiroaki Ida, Kenji Izuhara, Tomoaki Hoshino

**Affiliations:** 1 Division of Respirology, Neurology, and Rheumatology, Department of Internal Medicine, Kurume University School of Medicine, Ashahi-Machi 67, Kurume 830-0011, Japan; matama_goushi@med.kurume-u.ac.jp (G.M.);; 2 Department of Respirology and Clinical Research Center, National Hospital Organization Kyushu Medical Center, Jigyohama 1-8-1, Chuou-ku, Fukuoka 810-0065, Japan; 3 Department of Radiology, Kurume University School of Medicine, Ashahi-Machi 67, Kurume 830-0011, Japan; 4 Department of Radiology, Kansai Rosai Hospital, Inabasou 3-1-69, Amagasaki 660-0064, Japan; 5 Department of Respiratory Medicine, Nagasaki University Graduate School of Biomedical Sciences, Sakamoto 1-7-1, Nagasaki 852-8501, Japan; 6 Respiratory Medicine and Infectious Diseases, Oita University Faculty of Medicine, Idaigaoka 1-1, Hasama-Machi, Yufu 879-5593, Japan; 7 Department of Respiratory Medicine, Tenshindo Hetsugi Hospital, Nihongi 5956, Nakahetsugi 879-7761, Japan; 8 First Department of Internal Medicine, Shinshu University School of Medicine, Asahi 3-1-1, Matsumoto 390-8621, Japan; 9 Department of Pulmonary Medicine and Clinical Immunology, Dokkyo Medical University School of Medicine, Kitakobayashi 880, Mibu 321-0293, Japan; 10 Department of Respirology, Saga Medical School, Nabeshima 5-1-1, Saga 849-8501, Japan; 11 Division of Medical Biochemistry, Department of Biomolecular Sciences, Saga Medical School, Nabeshima 5-1-1, Saga 849-8501, Japan

**Keywords:** periostin, rheumatoid arthritis, interstitial lung disease, fibrosis, biomarker

## Abstract

Periostin was investigated as a biomarker for rheumatoid arthritis-associated interstitial lung disease (RA-ILD). This prospective study measured serum monomeric and total periostin, Klebs von den Lungen-6 (KL-6), surfactant protein D (SP-D), and lactate dehydrogenase (LDH) in 19 patients with RA-ILD, 20 RA without ILD, and 137 healthy controls (HC). All biomarkers were higher in RA-ILD than HC or RA without ILD. KL-6 accurately detected ILD in RA patients (area under curve [AUC] = 0.939) and moderately detected SP-D and monomeric and total periostin (AUC = 0.803, =0.767, =0.767, respectively). Monomeric and total periostin were negatively correlated with normal lung area and positively correlated with honeycombing, reticulation, fibrosis score, and the traction bronchiectasis grade but not inflammatory areas. Serum levels of SP-D, KL-6, and LDH did not correlate with the extent of those fibrotic areas on high-resolution CT. Serum monomeric and total periostin were higher in patients with RA-ILD with definite usual interstitial pneumonia pattern compared with other ILD patterns. Immunohistochemical analyses of biopsy or autopsy lung tissues from RA-ILD during the chronic phase and acute exacerbation showed that periostin was expressed in fibroblastic foci but not inflammatory or dense fibrosis lesions. Periostin is a potential biomarker for diagnosis, evaluating fibrosis, and deciding therapeutic strategies for patients with RA-ILD.

## 1. Introduction

Rheumatoid arthritis (RA) is an inflammatory autoimmune condition with a prevalence of approximately 0.5–1.0% of the general population [1]. The most significant extra-articular organ involvement in patients with RA is interstitial lung disease (ILD), which develops in approximately 1–67% of RA patients [2,3]. Risk factors for developing ILD were reported to be male gender, older age, obesity, smoking history, high titer of rheumatoid factor, and anti-cyclic citrullinated peptide antibody in patients with RA [2,3,4,5,6,7,8]. ILD is the major cause of death in patients with RA as well as malignant disease and the mortality rate of rheumatoid arthritis-associated interstitial lung disease (RA-ILD) is significantly higher than that of RA patients without ILD [4,5,6]. The reported median overall survival after diagnosis for RA-ILD was 2.6–3.0 years with a 5-year survival rate of 35–39% [5,6,7]. Factors reported predicting the mortality of RA-ILD were male gender, older age, lower forced vital capacity (FVC) and diffusing capacity of lungs for carbon monoxide (D_LCO_), wide areas of pulmonary fibrosis, usual interstitial pneumonia (UIP) pattern, presence of honeycombing on high-resolution computed tomography (HRCT), and developing acute exacerbation (AE) [6,7,8,9,10,11,12,13,14,15]. Enhancing the diagnosis of patients with early-stage ILD is an important issue for improving the prognosis of patients with RA [6,7,8]. The presence of ILD is usually diagnosed by chest HRCT in clinical practice [7,8]. However, performing chest HRCT examinations for all patients with RA is inconvenient. Some serum biomarkers such as Klebs von den Lungen-6 (KL-6) and surfactant protein D (SP-D) have superior diagnostic ability for patients with ILD but the evidence for predicting prognosis is insufficient [16,17,18,19,20,21]. Periostin is a matricellular protein that modulates cell matrix interactions via αvβ1, αvβ3, or αvβ5 integrin receptors [22]. Periostin is secreted from fibroblasts, epithelial cells, and endothelial cells after stimulation by interleukin (IL)-4, IL-13, and transforming growth factor-β and it contributes to the development of fibrosis in diseases such as systemic sclerosis and bronchial asthma [23,24]. We reported the upregulation of periostin in the lung tissues of mice with bleomycin-induced lung injury and its increased expression in the lungs and serum of human idiopathic interstitial pneumonias (IIPs) [25,26]. We and other groups have reported that high serum periostin levels were associated with decreased pulmonary function and shortened overall survival and time-to-event, including a greater than 5% decrease in the FVC, AE or death, and increased abnormal findings on HRCT in patients with idiopathic pulmonary fibrosis (IPF) [24,25,26]. Unfortunately, periostin is not a specific biomarker for ILD because it is upregulated in other diseases [23,24]. Izuhara et al. established a new enzyme-linked immunosorbent assay (ELISA) kit that specifically detects the monomeric form of periostin (SS20A × SS19D, capture and detection antibody). The level of monomeric periostin is more specific for IPF compared with that measured by conventional ELISA kits that detect the monomeric and oligomeric forms (SS18A × SS17B, total periostin) [27]. We showed that serum monomeric and total periostin were associated with decreases in the FVC and D_LCO_ in a multicenter prospective analysis [27]. Moreover, serum periostin was reported to be associated with the mortality of patients with fibrotic hypersensitivity pneumonia [28].

However, whether periostin is a biomarker in RA-ILD is unknown. Therefore, we retrospectively analyzed the performance of periostin as a biomarker to detect the presence of ILD and to evaluate the severity of disease in patients with RA.

## 2. Patients and Methods

### 2.1. Study Subjects

This study was conducted by the Consortium for Development of Diagnostics for Pulmonary Fibrosis Patients (CoDD-PF) study in seven hospitals from 2011 to 2014. Study samples were obtained from 39 patients with RA and 137 healthy controls who were enrolled in the CoDD-PF study. The study patients were recruited according to the following eligibility criteria: patients older than 20 years diagnosed with RA based on global guidelines were regarded as eligible patients [29]. Patients who developed AE within 3 months prior to enrollment were excluded from the study. The presence of ILD was diagnosed by investigators in each faculty based on the global guidelines and classification of IIPs [30,31]. A diagnosis of AE of ILD was defined by applying the criteria for IPF patients detailed in a previous report [32].

### 2.2. Study Protocol

Serum collection, evaluation of pulmonary function, and measurement of serum levels of lactate dehydrogenase (LDH), KL-6, and SP-D were performed at the time of subject enrollment. Chest HRCT examinations without contrast medium were performed at the baseline using a variety of scanners. The protocol of HRCT and diagnosis by radiological patterns applying the global guidelines or classification of IIPs, evaluation of the extent of disease, and HRCT features were performed as reported previously [25,27,30,31]. The protocol consisted of full inspiration in the supine position, with 0.5- to 1.5-mm collimation sections reconstructed with a high-spatial-frequency algorithm at 1-cm or 2-cm intervals. Images were interpreted at a window setting appropriate for viewing the lung parenchyma (window level, −600 to −700 Hounsfield units [HU]; window width, 1200 to 1500 HU). HRCT features evaluated in the present study included normal lung area, ground-glass attenuation (GGA), airspace consolidation, reticulation, and honeycombing. The inflammation score was defined as the sum of the extent of GGA and airspace consolidation and the fibrosis score was defined as that of reticulation and honeycombing. The grade of traction bronchiectasis (TBE) was quantified by assessing the levels of the most proximal bronchial branches involved. TBE was scored as follows: 0 = none, 1 = bronchial dilatation involving bronchi distal to the fifth generation, 2 = bronchial dilatation involving fourth-generation bronchi, and 3 = bronchial dilatation involving bronchi proximal to the third-generation bronchi. These TBE scores were assessed in each of the six lung zones and were averaged. Two board-certificated radiologists with 35 and 33 years of experience, who specialized in diffuse lung diseases with experience in chest CT interpretation, independently evaluated the HRCT findings. After assessing the interobserver agreement, the final decision was made by consensus. The radiologists were blinded to clinical information.

### 2.3. Measurement of Periostin by ELISA

Duplicated serum samples were obtained from subjects and then stored at −80 °C until human periostin ELISA was performed, which we established previously [25,27,28].

### 2.4. Immunohistochemical Assay

For immunohistochemical analysis, we investigated the expression of periostin in lung tissues obtained by surgical lung biopsy (SLB) and autopsy using rat anti-human periostin monoclonal antibodies (clone no. SS19B or SS5D) as reported previously [23,25]. We investigated the expression of periostin in lung tissues obtained from three patients with RA-ILD who underwent SLB at the chronic phase of the disease and who underwent autopsy after AE of ILD using anti-periostin monoclonal antibodies (0.1 μg/mL). Three patients with RA-ILD who underwent SLB at the chronic phase included a 56-year-old male, a 49-year-old female, and a 67-year-old male. Lung tissues were collected after AE from 3 RA-ILD patients who underwent autopsy including a 75-year-old male, 68-year-old male, and 69-year-old male. Two independent investigators with experience in the pathological diagnosis of ILD evaluated these sections without prior knowledge of the patients’ clinical status.

### 2.5. Statistical Analysis

Data are expressed as the median (25th–75th percentiles of the interquartile range). Differences between the two groups were analyzed as appropriate using the Wilcoxon rank-sum test or Fisher’s exact test. Multiple comparisons were correlated by Bonferroni’s method. Associations between the two groups were analyzed using Spearman’s rank correlation coefficient. The agreement between two independent observers was assessed using Cohen’s kappa statistics when classifying HRCT patterns by applying the global guidelines of IIPs [30,31]. A receiver operating characteristic (ROC) curve analysis was performed to evaluate the accuracy of differentiating RA-ILD patients from RA patients without ILD or healthy controls. An area under the ROC (AUC-ROC) from 0.50 to 0.70 represented poor accuracy, from 0.70 to 0.90 represented moderate accuracy, and higher values represented high accuracy. *p* < 0.05 was considered to represent statistical significance. The cut-off values were defined as the values with the highest Youden index (i.e., sensitivity + specificity − 1) on an ROC curve to distinguish patients with RA-ILD from controls. All statistical analyses were performed using JMP 17.0 (SAS Institute Japan, Tokyo, Japan).

## 3. Results

### 3.1. Patients’ Characteristics and Concordance of HRCT Evaluation between Two Investigators

**Figure 1:**
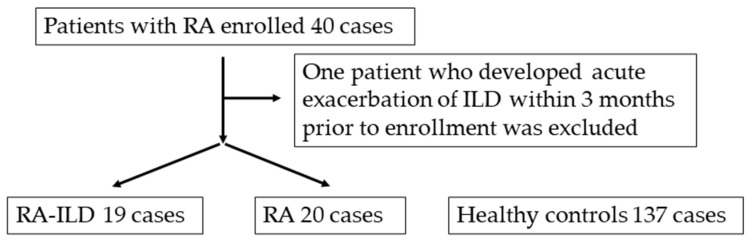
Study enrollment. RA, rheumatoid arthritis; ILD, interstitial lung disease.

The study enrollment is shown in Figure 1. Among 40 enrolled patients with RA, 1 patient who developed an AE of ILD within three months prior to enrollment was excluded from the study. The characteristics of all patients are shown in Table 1 and the pulmonary function and results of chest HRCT image evaluation of 19 RA-ILD patients are shown in Table 2. In total, 39 eligible RA patients including 10 (26%) males with a median age of 67 (60–74) years consisted of 19 (49%) with ILD and 20 (51%) without ILD. Overall, 137 healthy volunteers included 91 (66%) males and 64 (47%) smokers with a median age of 41 (30–50) years. Among 19 patients with RA-ILD, 6 (32%) patients were smokers but smoking status information among RA patients without ILD could not be obtained.

Serum levels of SP-D, KL-6, and monomeric and total periostin in RA-ILD patients were significantly higher than those in RA patients without ILD. The rates of female, age, and serum levels of LDH, SP-D, KL-6, and monomeric and total periostin in RA-ILD patients were significantly higher than those of healthy controls. The rates of female, age, and serum levels of LDH, KL-6, and monomeric periostin in RA patients without ILD were significantly higher than those in healthy controls.

The agreement between two independent observers for classifying HRCT patterns was excellent (kappa value, 0.91, *p* < 0.001). Similarly, the agreement between them regarding the extent of HRCT features was good to excellent (Spearman’s r, normal lung area, 0.91, GGA, 0.86, air space consolidation, 0.86, reticulation, 0.85; honeycombing, 0.95, inflammation score, 0.93, fibrosis score, 0.88; all *p* < 0.0001). There was no proportional bias or fixed bias in each Bland–Altman plot for the two parameters.

### 3.2. ROC Analysis to Differentiate RA-ILD Patients from RA Patients without ILD or Healthy Controls

**Figure 2:**
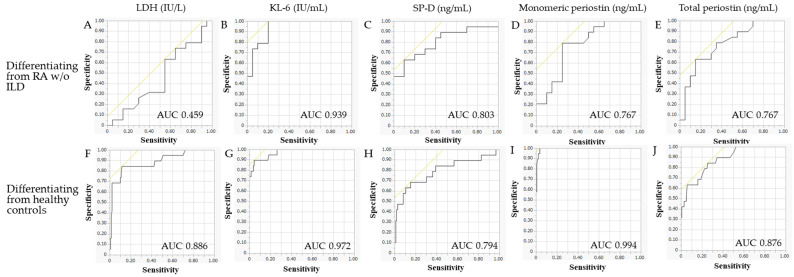
Receiver operating characteristic curves to differentiate RA-ILD patients from RA patients without ILD or healthy controls. Receiver operating characteristic curves to differentiate RA-ILD patients from RA patients without ILD and healthy controls by LDH (**A**), KL-6 (**B**), SP-D (**C**), monomeric periostin (**D**), and total periostin (**E**) are shown. Receiver operating characteristic curves to differentiate healthy controls from RA patients without ILD by LDH (**F**), KL-6 (**G**), SP-D (**H**), monomeric periostin (**I**), and total periostin (**J**) are shown. RA, rheumatoid arthritis; ILD, interstitial lung disease; LDH, lactate dehydrogenase; SP-D, surfactant protein D; KL-6, Klebs von den Lungen-6; AUC, area under receiver operating characteristic curve; *w*/*o*, without.

The results of the ROC analysis and optimal cut-off points and sensitivity and specificity to differentiate RA-ILD patients from RA patients without ILD or healthy controls are shown in Table 3 and Figure 2. The discriminatory power of KL-6 to detect the presence of ILD in RA patients was high (AUC, 0.939) and that of SP-D and monomeric and total periostin was moderate (AUC, 0.803, 0.767, and 0.767, respectively). Serum LDH levels could not detect the presence of ILD (AUC 0.459). The discriminatory power of KL-6 and monomeric periostin to differentiate RA-ILD patients from healthy controls was high (AUC, 0.972 and 0.994) and that of LDH, SP-D, and total periostin was moderate (AUC, 0.886, 0.794, and 0.876, respectively). The serum levels of SP-D, monomeric and total periostin, and especially KL-6 are potential diagnostic biomarkers for RA-ILD.

### 3.3. Correlation between Serum Biomarker Levels and Clinical Data of RA-ILD Patients

The results of an analysis to determine associations between serum biomarker levels and clinical data including pulmonary function and radiological findings on HRCT in RA-ILD patients using Spearman’s rank correlation coefficient are shown in Table 4 and Table 5. No biomarkers were associated with age. Analyses of associations between the analyzed serum biomarkers showed a correlation between serum levels of KL-6 and SP-D (r = 0.53) only. Analyses of associations between serum biomarkers and pulmonary function, other than the negative correlation (r = −0.53) between total periostin and vital capacity, showed that no other combinations had a significant correlation.

Analyses between serum biomarkers and the extent of an individual area on HRCT showed that monomeric and total periostin had a significant negative correlation with the extent of normal lung area (r = −0.57 and −0.62) and a positive correlation with the extent of honeycombing (r = 0.54 and 0.65), reticulation (r = 0.51 and 0.78), fibrosis score (r = 0.55 and 0.78), and TBE grade (r = 0.47 and 0.70), but not GGA, airspace consolidation, and inflammation score. A significant correlation was shown between SP-D and GGA (r = 0.54) and KL-6 and airspace consolidation (r = −0.47) but these two biomarkers did not correlate with other HRCT findings. None of the HRCT findings were associated with serum LDH levels.

Moreover, serum monomeric and total periostin levels were higher in 5 RA-ILD patients showing a definite UIP pattern on HRCT compared with 14 patients showing possible UIP or inconsistent with UIP patterns. There were no significant differences between the serum levels of LDH, SP-D, and KL-6 between definite UIP and other ILD patterns on HRCT. In the present study, serum periostin levels, but not other analyzed biomarkers, were associated with the extent of radiological fibrotic involvement in RA-ILD patients. In contrast, serum periostin levels were not associated with radiological inflammatory involvement in RA-ILD patients.

### 3.4. Immunohistochemical Assay Using Anti-Periostin Monoclonal Antibodies

**Figure 3:**
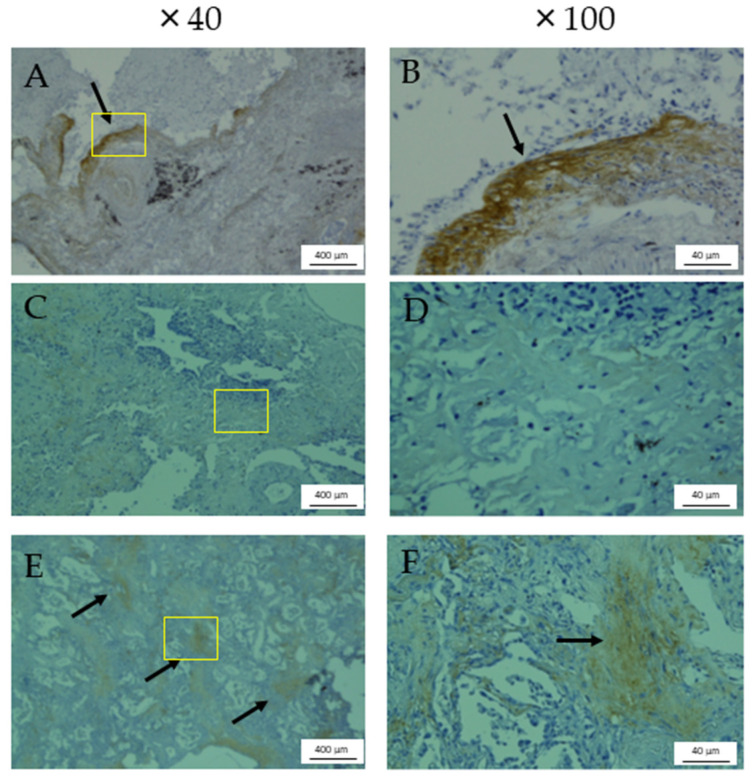
Immunohistochemical assay using anti-periostin monoclonal antibodies. Immunohistochemical assay of lung tissues obtained from representative RA-ILD patients by surgical lung biopsy (male, 56 years, (**A**,**C**), ×40; (**B**,**D**), ×100) and acute exacerbation of RA-ILD by autopsy (male, 75 years, (**E**), ×40; (**F**), ×100) using anti-periostin monoclonal antibodies (0.1 μg/mL). Periostin expression was observed in fibroblastic foci (arrows, (**A**,**B**,**E**,**F**)) but not in lesions of inflammation or dense fibrosis (**C**,**D**). The yellow box shows the area of high-power field.

Periostin expressions in lung tissues obtained from a representative male patient with RA-ILD who underwent SLB at 56 years of age and a male patient with RA-ILD who underwent an autopsy after AE at 75 years of age are shown in Figure 3. Periostin expression was observed in fibroblastic foci (Figure 3A,B,E,F) but not in lesions of inflammation or dense fibrosis (Figure 3C,D).

## 4. Discussion

In the present study, we demonstrated that serum monomeric and total periostin can be used for the diagnosis and evaluation of fibrotic involvement in RA-ILD patients.

The efficacy of KL-6 and SP-D as well as monomeric and total periostin as diagnostic biomarkers for RA-ILD was demonstrated and KL-6 had the highest ROC-AUC for differentiating RA-ILD patients from RA patients without ILD or healthy controls. Previous studies reported that KL-6 detected the presence of ILD in RA patients [19,20]. Moreover, KL-6 was positively correlated with a semi-quantitative grade of ILD extent, negatively correlated with the FVC and D_LCO_ in connective tissue disease-associated ILD [20], and associated with the short-term progression and survival of RA-UIP [21]. The efficacy of SP-D as a biomarker for systemic sclerosis-associated ILD was reported but its use for RA-ILD is unknown [33,34]. Lee et al. suggested that the addition of KL-6 and IL-6 predicted short-term disease progression in RA-UIP patients better than a single biomarker [21]. Combinations of multiple biomarkers can supplement chest HRCT examination for the diagnosis of early-stage ILD in RA patients in clinical practice. In the present study, why the serum levels of LDH, KL-6, and monomeric periostin in RA patients without ILD were significantly higher than those in healthy controls was not clear. However, the median serum levels of LDH and KL-6 were lower than their cut-off levels. Higher levels of monomeric periostin in RA patients without ILD may be explained by previous studies in which the expression of periostin was increased in serum, synovium tissue, and synovial fluid of RA patients compared with healthy controls or osteoarthritis patients [35,36]. Due to the fact that elevated periostin levels in RA patients without ILD cause false positive diagnoses with ILD, the final ILD diagnosis in RA patients with high periostin levels should be performed by HRCT.

In the present study, serum monomeric and total periostin levels were inversely correlated with the semi-quantitative extent of normal lung area and positively correlated with fibrotic involvement and traction bronchiectasis grade but not inflammatory involvement on chest HRCT. Monomeric and total periostin levels were higher in RA-ILD patients with a definite UIP pattern on HRCT compared with other ILD patterns. SP-D and KL-6 levels were correlated with the extent of GGA or airspace consolidation but not correlated with other HRCT findings. We previously suggested that the serum total periostin level was associated with an increase in the extent of honeycombing on HRCT scans of IPF patients [24]. In the present study, periostin was also associated with the severity of radiological fibrotic involvement in RA-ILD patients compared with other biomarkers. The extent of lung involvement on HRCT or radiological UIP pattern was associated with the survival of patients with RA-ILD [9,11,12,13]; thus, periostin may be a potential prognostic factor for RA-ILD patients as well as IPF patients, as we reported previously [24,25,27]. Further study is required to clarify this issue. Our immunohistochemical assay demonstrated that periostin was expressed in fibroblastic foci but not inflammatory or dense fibrosis lesions. We previously reported an immunohistochemical semi-quantitative analysis showing that periostin was strongly expressed in the lungs of IPF and fibrotic nonspecific interstitial pneumonia (NSIP) patients but weakly expressed in cellular NSIP and cryptogenic organizing pneumonia patients as well as normal lungs [25]. The expression of periostin was observed in fibroblasts, especially in fibroblastic areas, but not in regenerative alveolar epithelium or macrophages, areas with established fibrosis, or inflammatory cells [25]. Periostin was also specifically expressed in active fibrotic lesions during AE of RA-ILD patients. Moreover, Shimizu et al. reported that monomeric periostin was associated with survival after AE in fibrotic ILD patients [37]. Therefore, the high expression of periostin in the fibrotic lesions of RA-ILD patients in the present study may be related to disease progression, which should be analyzed further.

Biomarkers specifically associated with fibrotic involvement might contribute to determining therapeutic strategies for ILD, particularly the start of antifibrotic drug therapy. In RA-ILD patients, the pathologic targets for drug therapy are pulmonary inflammation or fibrosis. Treatment with anti-inflammatory drugs such as immunosuppressants should be selected first to suppress synovitis and/or systemic inflammation and, if progressive lung fibrosis develops, antifibrotic drugs should be added [14,15]. The addition of antifibrotic drugs should be initiated early in cases with a radiological UIP pattern or large fibrotic involvement [14,15]. A randomized controlled phase 3 (INBUILD) trial reported that treatment with nintedanib, an antifibrotic drug, had efficacy in progressive pulmonary fibrosing ILD patients including those with RA-ILD [38]. Since nintedanib therapy for ILD patients with a radiological UIP pattern had a more favorable therapeutic response than in those with a non-UIP pattern in that trial [38], classifying whether the radiological ILD pattern is UIP is important in clinical practice. However, the classification of radiological ILD patterns based on global IIP guidelines is more difficult than IIPs and they are often classified as indeterminate for UIP patterns in patients with RA-ILD because of the coexistence of various pathological lung involvements such as airway disease [30,31,39,40]. The evaluation of periostin as a lung fibrosis biomarker in combination with HRCT examination may contribute to deciding therapeutic strategies for RA-ILD patients whom it is difficult to classify by radiological ILD patterns.

The present study had some limitations. First, the sample size of the present study was small. In particular, a major limitation of the immunohistochemical assays of tissues from RA-ILD patients was that they were only performed using a small number of patients. Since the biomarker expression in lung tissues of RA-ILD patients are novel and important, immunohistochemical assays using a larger number of patients is needed. Second, in the analysis of diagnostic biomarkers for RA-ILD, statistical adjustment for other risk factors of developing ILD such as gender, age, and smoking history was not possible because of the small number of subjects. In addition, information on smoking history was not available for RA patients without ILD. Third, we were unable to analyze the association between serum biomarkers and clinical outcomes, such as mortality and disease progression, because of the small number of cases in which the clinical course could be observed. Further cohort studies with a large population and long-term observation are needed to analyze these issues.

## 5. Conclusions

The serum periostin level is a potential biomarker for the diagnosis of RA-ILD and for evaluating fibrotic involvement in RA-ILD patients. The evaluation of periostin as a lung fibrosis biomarker in combination with HRCT examination may contribute to deciding therapeutic strategies for RA-ILD patients.

## Figures and Tables

**Table 1:** Patients’ characteristics.

				*p* Value			
				Among	RA-ILD vs.	RA-ILD vs.	RA *w*/*o* ILD
	RA with ILD	RA *w*/*o* ILD	Healthy Control	3 Groups	RA *w*/*o* ILD	Control	vs. Control
N	19	20	137				
Age	70 (65–76)	66 (60–73)	41 (30–50)	<0.0001 *	0.14	<0.0001 *	<0.0001 *
Male gender	6 (32%)	4 (20%)	91 (66%)	<0.0001 *	0.48	0.0050 *	0.0001 *
Smoker	6 (32%)	N.D.	64 (47%)			0.23	
LDH (IU/L)	205.0 (178.0–233.0)	195.0 (164.3–232.8)	149.0 (136.0–164.5)	<0.0001 *	0.66	<0.0001 *	<0.0001 *
SP-D (ng/mL)	105.0 (50.2–203.0)	36.1 (22.8–69.5)	40.5 (26.7–66.3)	0.0002 *	0.0012 *	<0.0001 *	0.72
KL-6 (IU/mL)	1022.0 (579.0–1467.0)	214.5 (177.3–273.0)	274.0 (226.5–336.0)	<0.0001 *	<0.0001 *	<0.0001 *	0.0048 *
Monomeric periostin (pg/mL)	15.9 (14.3–20.1)	13.4 (10.8–16.3)	8.3 (7.2–9.9)	<0.0001 *	0.0043 *	<0.0001 *	<0.0001 *
Total periostin (pg/mL)	100.0 (77.0–140.0)	73.5 (55.8–84.8)	63.0 (52.5–75.5)	<0.0001 *	0.0035 *	<0.0001 *	0.10

Data are expressed as the median (25th–75th percentiles of the interquartile range). RA, rheumatoid arthritis; ILD, interstitial lung disease; LDH, lactate dehydrogenase; SP-D, surfactant protein D; KL-6, Klebs von den Lungen-6; *w*/*o*, without; N.D., no data; * significant.

**Table 2:** Pulmonary function and results of evaluating chest HRCT images of 19 RA-ILD patients.

Pulmonary Function	
Vital capacity (%)	84.4 (66.7–107.3)
D_LCO_ (%)	71.4 (60.7–86.1)
HRCT pattern	
Definite UIP	5 (25%)
Possible UIP	3 (15%)
Inconsistent with UIP	12 (60%)
Extent of abnormal area on chest HRCT (%)	
Normal lung area	137.5 (100.0–155.0)
Honeycombing	0 (0–7.5)
Reticulation	25.0 (10.0–40.0)
Ground-glass attenuation	22.5 (20.0–47.5)
Airspace consolidation	0 (0–10.0)
Fibrosis score	25.0 (10.0–40.0)
Inflammation score	32.5 (20.0–50.0)
TBE grade on HRCT	13.0 (7.0–30.0)

HRCT, high-resolution computed tomography; RA, rheumatoid arthritis; ILD, interstitial lung disease; D_LCO_, diffusing capacity of lungs for carbon monoxide; UIP, usual interstitial pneumonia; TBE, traction bronchiectasis; Inflammation score, sum of the extent of GGA and airspace consolidation; Fibrosis score, the sum of reticulation and honeycombing.

**Table 3:** Receiver operating characteristic curve analysis to differentiate RA-ILD patients from RA patients without ILD or healthy controls.

	AUC	Standard Error	*p* Value	Cut-Off Value	Sensitivity (%)	Specificity (%)
Distinguishing RA without ILD patients						
LDH (IU/L)	0.459	0.0028	0.53	218.0	73.7	35.0
KL-6 (IU/mL)	0.939	0.0020	<0.0001 *	329.0	100.0	80.0
SP-D (ng/mL)	0.803	0.0088	0.0002 *	93.9	63.2	90.0
Monomeric periostin (ng/mL)	0.767	0.081	0.0089 *	14.3	79.0	75.0
Total periostin (ng/mL)	0.767	0.013	0.0027 *	98.0	63.2	85.0
Distinguishing Healthy controls						
LDH (IU/L)	0.886	0.0096	<0.0001 *	177.0	84.2	88.3
KL-6 (IU/mL)	0.972	0.0032	<0.0001 *	423.0	89.5	95.6
SP-D (ng/mL)	0.794	0.0050	<0.0001 *	79.5	68.4	87.6
Monomeric periostin (ng/mL)	0.994	0.443	<0.0001 *	12.5	100.0	96.3
Total periostin (ng/mL)	0.876	0.0126	<0.0001 *	75.0	84.2	74.0

RA, rheumatoid arthritis; ILD, interstitial lung disease; LDH, lactate dehydrogenase; SP-D, surfactant protein D; KL-6, Klebs von den Lungen-6; AUC, area under receiver operating characteristic curve; * significant.

**Table 4:** Correlation between serum biomarker levels and clinical data.

	Monomeric Periostin (ng/mL)		Total Periostin (ng/mL)		KL-6 (IU/mL)		SP-D (ng/mL)		LDH (IU/L)	
	R	*p* Value	R	*p* Value	R	*p* Value	R	*p* Value	R	*p* Value
Age (years)	0.17	0.50	0.26	0.28	−0.069	0.78	−0.0053	0.98	0.19	0.44
Serum biomarkers										
SP-D (ng/mL)	0.073	0.77	0.12	0.61	0.53	0.019 *	-	-	0.24	0.33
LDH(IU/L)	−0.30	0.22	0.078	0.75	0.36	0.13	0.24	0.33	-	-
KL-6 (IU/mL)	0.23	0.35	0.29	0.23	-	-	0.53	0.019 *	0.36	0.13
Pulmonary function										
Vital capacity (%)	−0.32	0.19	−0.53	0.022 *	−0.063	0.80	−0.35	0.16	−0.21	0.39
D_LCO_ (%)	−0.18	0.52	−0.38	0.16	−0.50	0.060	−0.45	0.090	−0.31	0.27
Extent of abnormal area on chest HRCT (%)										
Normal lung area	−0.57	0.011 *	−0.62	0.0045 *	−0.44	0.062	−0.35	0.15	−0.13	0.58
Honeycombing	0.54	0.018 *	0.65	0.0025 *	0.28	0.25	−0.12	0.64	−0.12	0.64
Reticulation	0.51	0.013 *	0.78	0.0002 *	0.18	0.47	0.045	0.85	0.18	0.47
GGA	−0.031	0.90	−0.14	0.56	0.44	0.061	0.54	0.017 *	0.25	0.31
Airspace consolidation	0.19	0.44	0.069	0.78	−0.47	0.041 *	−0.26	0.29	−0.41	0.081
Fibrosis score	0.55	0.014 *	0.78	<0.0001 *	0.18	0.47	0.051	0.83	0.11	0.65
Inflammation score	−0.091	0.71	−0.13	0.59	0.25	0.31	0.31	0.19	0.14	0.56
TBE grade on HRCT	0.47	0.041 *	0.70	0.0008 *	0.33	0.16	0.41	0.079	0.19	0.42

HRCT, high-resolution computed tomography; D_LCO_, diffusing capacity of lungs for carbon monoxide; LDH, lactate dehydrogenase; SP-D, surfactant protein D; KL-6, Klebs von den Lungen-6; GGA, ground-glass attenuation; TBE, traction bronchiectasis; Inflammation score, the sum of the extent of GGA and airspace consolidation; Fibrosis score, the sum of reticulation and honeycombing; * significant.

**Table 5:** Association between biomarkers and radiological ILD patterns.

	Definite UIP Pattern		*p* Value
	Yes	No	
N	5	14	
LDH (IU/L)	200.0 (163.0–218.0)	207.5 (180.3–262.8)	0.19
KL-6 (IU/mL)	1414.0 (554.5–1905.0)	942.5 (541.8–1367.3)	0.52
SP-D (ng/mL)	93.9 (35.9–240.0)	123.0 (56.7–212.0)	0.52
Monomeric periostin (pg/mL)	20.1 (18.6–50.5)	15.3 (13.5–17.5)	0.018 *
Total periostin (pg/mL)	143.0(121.9–202.5)	90.0 (73.8–115.0)	0.0095 *

ILD, interstitial lung disease; UIP, usual interstitial pneumonia; LDH, lactate dehydrogenase; SP-D, surfactant protein D; KL-6, Klebs von den Lungen-6; * significant.

## Data Availability

The data presented in this study are available on request from the corresponding author. The data are not publicly available due to ethical considerations.
